# Anatomy-Aware Inference of the 3D Standing Spine Posture from 2D Radiographs †

**DOI:** 10.3390/tomography8010039

**Published:** 2022-02-11

**Authors:** Amirhossein Bayat, Danielle F. Pace, Anjany Sekuboyina, Christian Payer, Darko Stern, Martin Urschler, Jan S. Kirschke, Bjoern H. Menze

**Affiliations:** 1 Department of Computer Science, Technical University of Munich, 85748 Garching, Germany; anjany.sekuboyina@tum.de (A.S.); bjoern.menze@uzh.ch (B.H.M.); 2 Department of Neuroradiology, Klinikum rech der Isar, 81675 Munich, Germany; jan.kirschke@tum.de; 3 Computer Science and Artificial Intelligence Laboratory, Massachusetts Institute of Technology, Cambridge, MA 02139, USA; dfpace@mit.edu; 4 A.A. Martinos Center for Biomedical Imaging, Massachusetts General Hospital, Harvard Medical School, Boston, MA 02114, USA; 5 Department of Quantitative Biomedicine, University of Zurich, 8006 Zurich, Switzerland; 6 Institute of Computer Graphics and Vision, Graz University of Technology, 8010 Graz, Austria; christian.payer@icg.tugraz.at (C.P.); stern@icg.tugraz.at (D.S.); 7 School of Computer Science, University of Auckland, Auckland 1010, New Zealand; martin.urschler@auckland.ac.nz

**Keywords:** 3D reconstruction, shape priors, neural networks, registration, template

## Abstract

An important factor for the development of spinal degeneration, pain and the outcome of spinal surgery is known to be the balance of the spine. It must be analyzed in an upright, standing position to ensure physiological loading conditions and visualize load-dependent deformations. Despite the complex 3D shape of the spine, this analysis is currently performed using 2D radiographs, as all frequently used 3D imaging techniques require the patient to be scanned in a prone position. To overcome this limitation, we propose a deep neural network to reconstruct the 3D spinal pose in an upright standing position, loaded naturally. Specifically, we propose a novel neural network architecture, which takes orthogonal 2D radiographs and infers the spine’s 3D posture using vertebral shape priors. In this work, we define vertebral shape priors using an atlas and a spine shape prior, incorporating both into our proposed network architecture. We validate our architecture on digitally reconstructed radiographs, achieving a 3D reconstruction Dice of 0.95, indicating an almost perfect 2D-to-3D domain translation. Validating the reconstruction accuracy of a 3D standing spine on real data is infeasible due to the lack of a valid ground truth. Hence, we design a novel experiment for this purpose, using an orientation invariant distance metric, to evaluate our model’s ability to synthesize full-3D, upright, and patient-specific spine models. We compare the synthesized spine shapes from clinical upright standing radiographs to the same patient’s 3D spinal posture in the prone position from CT.

## 1. Introduction

**Figure 1:**
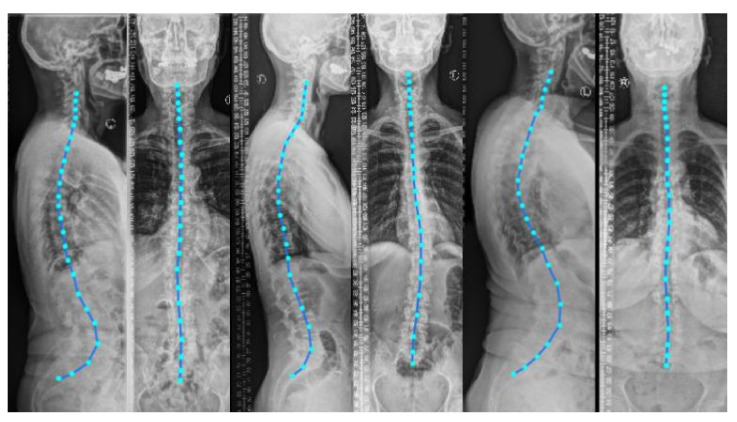
Lateral and anterior-posterior (AP) view radiographs of three patients with spinal curvature annotation. Considering the vertebral centroid coordinates and spinal curvature facilitates determining the scale and the vertebral orientation.

A biomechanical load analysis of the spine in an upright standing position is highly warranted in various spine disorders to understand their cause and guide therapy [1]. Typical approaches for load estimation either use a computational shape model of the spine for all patients or obtain a subject-specific spine model from a 3D imaging modality such as magnetic resonance imaging (MRI) or computed tomography (CT) [2]. Even though MRI and CT images can capture 3D anatomical information, they need the patient to be in a *prone* or *supine* position (lying flat on a table) during imaging. Nevertheless, to analyze the spinal alignment in a physiologically upright standing position under weight bearing, orthogonal 2D plain radiographs (as depicted in Figure 1) are the *de facto* choice. A combination of both these worlds is of clinical interest to fully assess the true biomechanical situation, that is, to capture the patient-specific complex pathological spinal arrangement in a standing position with full 3D information [2,3,4].

As crucial spatial information is lost when projecting a 3D object in only two 2D planes [5], a random object cannot reliably be reconstructed from two orthogonal projections. However, the spine follows strong anatomical rules, that are repeated only with slight variations in any patient. Typical projections—that is, lateral and AP radiographs—cover most of these variations, both on a local (per vertebra) and global (overall spinal alignment) level.

The literature offers a wealth of registration-based methods [4,6,7,8,9,10] for relating 2D radiographs to 3D CT or MR images. In [4], the authors employ coarse manual registration for aligning 3D data to 2D sagittal radiographs for the lumbar vertebrae. Similarly, in [6], manually annotated vertebral bodies on 2D radiographs are used to measure the vertebral orientations in an upright (standing) position. Such solutions are time-consuming, labor-intensive, and are vulnerable to error. Note that both works only employ sagittal reformations to position the vertebrae. They thus ignore coronal reformation containing significant information about the spine’s natural curvature, especially in abnormal cases. Other approaches for this purpose introduce an automated 3D–2D spine registration algorithm [7,8], wherein authors suggest a multi-stage registration method by introducing a comparison metric for a CT projection and a radiograph. Since this metric is parameter-heavy and hand-crafted, the generalizability and inference speeds of these methods are limited. In [9,10], the 3D shape of the spine is reconstructed using a biplanar X-ray device called ‘EOS’. The advantage of the system is the low radiation dose required and that both projections are acquired simultaneously, allowing for a direct spatial correspondence between the two planes. Hindering its applicability is the high device cost and thus the lack of its presence in clinical routines.

The problem of reconstructing 3D shapes from 2D images has recently been explored using deep learning methods. For example, in [11], adversarial training is used to synthesize 3D CT images given two orthogonal radiographs. For a scale of spinal scans, this method is memory intensive and also fails to synthesize smaller 3D anatomies such as the vertebrae. Note that this method has been evaluated on digitally reconstructed radiographs (DRR) only, thus requiring further validation for its clinical usage. In [12,13], the authors design a model to generate a 3D shape given multiple arbitrary view 2D images. However, the input images include only the object of interest without background, which is not applicable to medical images like spinal radiographs. In our previous work on inferring the 3D standing spine posture [14], we introduce a new deep neural network architecture termed TransVert, to combine the 2D orthogonal image information and reconstruct a 3D shape. Since the vertebrae are heavily occluded by soft tissue and ribs in the radiographs, the reconstruction is challenging and in some cases the output shape is far from a vertebral shape.

In machine learning, shape priors help to reduce the search space of possible solutions, improving the accuracy and plausibility of solutions [15]. Priors are particularly effective when data are unclear, corrupt, with low signal-to-noise ratio or when training data are scarce [15,16]. The idea of incorporating shape priors into the neural network models has been explored by [15,17,18,19,20]. Most of the ideas proposed for using shape priors in deep neural networks are for a single object, while the spine shape is more complicated as it is made of multiple objects (vertebrae) connected to each other. The spine and vertebrae shapes are deformed, subject to some constraints of human anatomy. Thus, defining a prior for explaining intervertebral constraints and the spinal shape is crucial here.

A few works have modeled the spinal shape [10,21,22,23,24]. For instance, in [24] the authors proposed an automatic framework that segments vertebrae from arbitrary CT images with a complete spine model. They first scanned a commercially available plastic phantom to generate the template. Next, they manually registered it to ten actual full spine scans. The authors learned a statistical shape model of the spine in [23] by independently studying three models for cervical, thoracic and lumbar regions. Thus, their models do not learn the shape correlations across the full spine [21]. In [22], the authors propose a parametric model of the spine, which is computed using statistical inferences, image analysis techniques and manual rigid registration. In [10], the authors used a PCA-based model to decompose the spine shape into the spinal curve and local shape of vertebrae.

Besides statistical shape models [10,21,23,24], other probabilistic models, such as probabilistic atlas [25], graph models [26], Hidden Markov Models [27] and hierarchical models [28,29,30] have also been proposed. For instance, in [25], the authors proposed a probabilistic atlas of the spine. By co-registering 21 CT scans, a probability map is created which can be used to segment and detect the vertebrae with a special focus on ribs suppression. In [26] the authors proposed a probabilistic graphical model for the location and identification of the vertebrae in MR images. In both cases full spines were observed at training time. However, none of the works mentioned above are an end-to-end machine learning treatment of the problem of the reconstruction of 3D shapes of spines from 2D images.

We introduce an anatomy-aware deep neural network for fusing two orthogonal 2D radiographs to generate a 3D spine model. We define this 3D shape synthesis as a hybrid registration problem in which the network estimates vector fields to deform vertebral shape templates to achieve shape synthesis. We apply our model to clinical radiographs subject to noise and heavy tissue overlay. We incorporate shape priors into the model to enhance robustness against such artefacts in real-world data. We propose a training approach using synthetically generated radiographs from 3D CT, with full supervision from the CT’s 3D vertebral masks.

## 2. Materials and Methods

For synthesizing 3D data from 2D information, the following requirements are desired: First, for efficient recovery of 3D shape information from sagittal and coronal projections, the network needs to integrate the information from these projections appropriately. Second, recovering 3D shapes from 2D projections is inherently an ill-posed problem, requiring incorporation of prior knowledge. This knowledge includes vertebral shapes and the shape of the spine (spinal curvature).

Our work is mainly based on the TransVert network [14]. The TransVert network is designed for inferring the 3D standing spinal posture from 2D radiograph. Here, we overview the TransVert model to better explain our proposed model. We will introduce the TransVert+ model to address the limitations of TransVert.

### 2.1. TransVert

**Figure 2:**
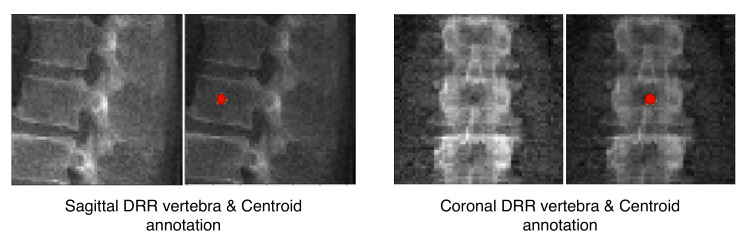
Vertebral image patches with corresponding annotations. The network inputs are 2D orthogonal view vertebrae patches and the centroid indicates the vertebra of interest.

TransVert inputs are the sagittal and coronal vertebral image patches and their corresponding annotation images. The annotation images indicate the vertebra-of-interest (VOI). Given the four 2D inputs, the model predicts the vertebra’s full-body 3D shape, y, as a discrete voxel-map:

(1) $$y=G(x_{s},x_{c},y_{s},y_{c}),$$

where *G* denotes the mapping performed by TransVert, $x_{s}$ and $x_{c}$ denote the 2D vertebral sagittal and coronal reformations; $y_{s}$ and $y_{c}$ denote the corresponding VOI annotations. The VOI-annotation image is obtained by placing a disc of radius 1 mm around the vertebral centroid. Figure 2 depicts orthogonal input image patches with corresponding centroid annotations, indicating the vertebra of interest in that patch. In [14], other annotation choices (vertebral body and full vertebral masks) are analyzed in several experiments. The TransVert model is trained on sagittal and coronal digitally reconstructed radiographs (DRR) created from CT images. The model is supervised by the corresponding CT images’ voxel-level, vertebral segmentation masks. Since DRRs are similar in appearance to real radiographs, a model trained on DRRs can be deployed on clinical radiographs.

**Figure 3:**
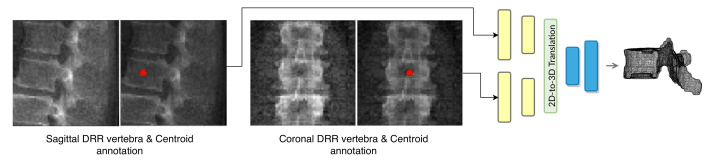
Overview of 2D image to 3D shape translation in TransVert. The network inputs are 2D orthogonal view vertebrae patches and the centroid indicating the vertebra of interest.

Figure 3 overviews the TransVert architecture [14]. TransVert consists of three blocks: a 2D sagittal encoder, a 2D coronal encoder, and a 3D decoder. In [14] we introduced a ‘map&fuse’ mechanism to combine the three blocks. The map&fuse block is designed to map 2D representation of the sagittal and coronal views into intermediate 3D latent representations and fuse them into a single 3D representation. The decoder block maps the 3D latent representation to a 3D shape in voxel space.

### 2.2. TransVert+: Anatomy-Aware TransVert

The TransVert model [14] performs 2D to 3D translation vertebra by vertebra. While this results in acceptable vertebral shape reconstruction, the model still is not aware of the spinal shape and relative position of vertebrae, which might result in inconsistency in the reconstructed spinal shape. To overcome this problem, we propose incorporating the spinal curve and vertebral shapes as prior. Instead of regressing the shapes directly, our model deforms an atlas to the desired vertebral shape.

Our network estimates the vector field that deforms a discrete atlas to the desired vertebral shape. We incorporate a spine atlas as a shape prior into our network architecture, to enforce vertebral shape constraints. We also define the spine curvature by atlas vertebral centroids, for enforcing the shape of the spine. In addition to image data, we include the vertebral labels as an additional annotation attached to the network input as depicted in Figure 2. We take a registration approach for shape synthesis and propose the TransVert+ network architecture.

TransVert+ takes five inputs, Equation (2), of which four are 2D images, the same as TransVert’s inputs, depicted in Figure 2. The fifth input is a vector of floats denoting the coordinates of the vertebral centroids (${\vec{C}}^{\;v}$) in a global spine coordinate system, for providing the model with a holistic view of the spinal curve for more consistency. We desire a function *G* that outputs the vertebra’s full-body 3D shape, y, which is represented as a discrete voxel-map by deforming the vertebral shape templates ($y_{t}$):

(2) $$y=G\left(x_{s},x_{c},y_{s},y_{c},{\vec{C}}^{\;v}\right)\circ y_{t}.$$

We formulate this shape synthesis as a registration problem in which vertebral shape templates are deformed to obtain desired shapes. This includes both global affine transformations (scaling and 3D rotation for each vertebra, considering the global spinal shape) and local deformations on the vertebral surface. We denote them as two sub-tasks,

(3) $$G\left(x_{s},x_{c},y_{s},y_{c},{\vec{C}}^{\;v}\right)=G_{a}+G_{d},$$

(4) $$G_{a}=AffineDecoder\left(x_{s},x_{c},y_{s},y_{c},{\vec{C}}^{\;v}\right),$$

(5) $$G_{d}=DeformableDecoder\left(x_{s},x_{c},y_{s},y_{c}\right),$$

where *G* in Equation (3) denotes the mapping performed by TransVert+. The transformation *G* is separated into affine ($G_{a}$) and deformable ($G_{d}$) transformations, explained in Equations (4) and (5) respectively. When inferring the affine transformation the entire spinal shape is considered, while for inferring the deformable transformation individual vertebral shape features are taken into account. We will elaborate more on this in the next sections.

Ideally, training the TransVert+ model requires radiograph images and their corresponding ‘real world’ standing 3D spine models. However, this correspondence does not exist. It is, in effect, the issue we aim to address. Thus, TransVert+ is trained on sagittal and coronal digitally reconstructed radiographs (DRR) generated from prone CT images with supervision from the voxel-level, vertebral segmentation masks of the corresponding CT images.

Generating 3D shapes from 2D information is an ill-posed problem. We model this task as a registration problem. Given 2D orthogonal information we estimate vector fields to deform shape templates to match the target 3D shape. We introduce a novel deep neural architecture to fuse the 2D information from orthogonal views and estimate the vector field required to maximize the alignment of the deformed template and the target shape. More specifically, to infer 3D spinal shapes, given 2D orthogonal radiographs and vertebral centroid coordinates in the global coordinate system, our model predicts a vector field to deform each vertebral shape template to match the target. The vector fields are predicted by considering each vertebral shape locally and also considering the spinal curvature globally. We show that incorporating the global spinal shape information improves the performance of the model.

Our model is composed of two encoders for each view, an affine decoder and a deformable decoder. In the following sections, we describe the model architecture and training scheme in detail.

### 2.3. Network Architecture

**Figure 4:**
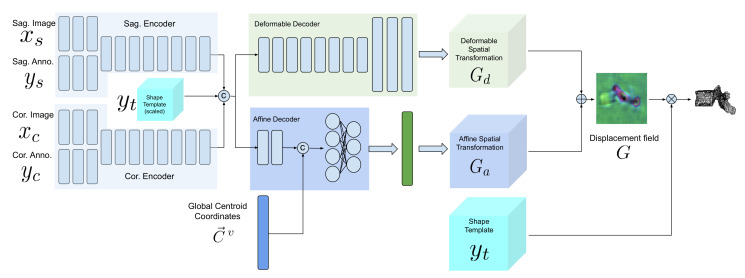
Architecture of TransVert+. Our model is composed of sagittal and coronal 2D encoders, an affine 3D decoder and a deformable 3D decoder. The down-scaled shape templates are concatenated with the features extracted by encoders and fed to the decoders. The centroid coordinates in the global spine coordinate system are concatenated with the affine decoder feature maps. The ⊗, ⊕ and © operators represent warping, addition and concatenation, respectively.

Figure 4 demonstrates a block diagram of the model and its subnetworks. The model is composed of two encoders, one for each view (a sagittal encoder and a coronal encoder) and two decoders (an affine decoder and a deformable decoder). The input to the encoders is the image patch of the vertebra from the radiograph and the centroid annotation on the vertebra of interest. The features extracted using the encoders are concatenated to the global centroid coordinates and fed to the affine decoder (to estimate the affine transformation parameters for each vertebra) and the deformable decoder (which is a fully convolutional network to estimate the deformation for each voxel). Next, the affine and deformable vector fields are summed to produce the final displacement field, which is used to warp the template and produce the 3D shape model. Finally, a 3D model of the spine can be generated by stacking the predicted 3D vertebrae shapes at their corresponding 3D centroid locations.

#### 2.3.1. Sagittal and Coronal Encoders

**Figure 5:**
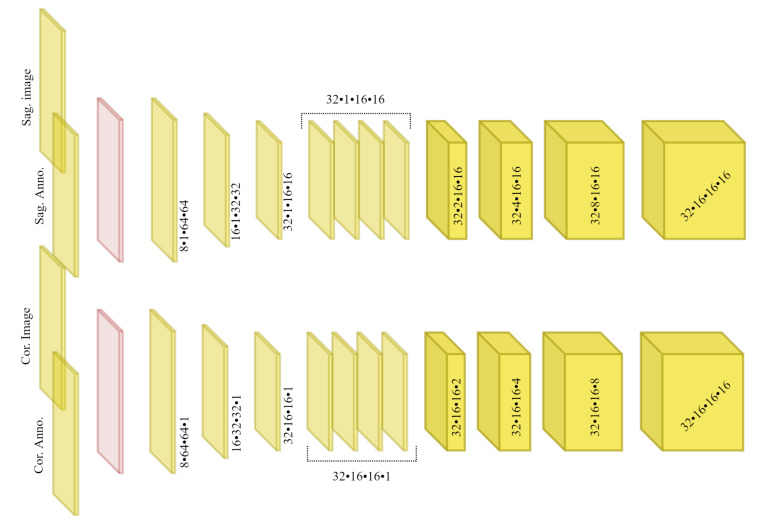
Architecture of the orthogonal encoders, which employ anisotropic convolutions, with an anisotropy along the dimensions that need to be expanded. We use ‘squeeze and excitation’ blocks (depicted in red) to fuse the image features and annotation features.

The architecture of sagittal and coronal encoders are designed differently. In Figure 5, the architecture for each view is visualized. Each encoder is designed to reconstruct the missing third dimension of the 2D input. Therefore, they contain anisotropic convolutions with the longer side along the dimension that needs to be expanded. For instance, for a coronal input the anterior–posterior dimension needs to be expanded. For the same reason, the convolutional strides and padding directions are orthogonal for each of the views. We empirically observed that, employing the ‘squeeze and excitation’ block in the network results in a better performance than a naive fusion by concatenating the multiple channels. As input to the encoders, the vertebral images and VOI-annotations are combined using a ‘squeeze and excitation’ block (depicted in red) [31]. In our previous work [14] we showed that, using encoders for each view with anisotropic convolutions, outperforms using a single encoder or fusing input 2D data by outer product of the orthogonal 2D images.

#### 2.3.2. Affine Decoder

Since the vertebrae are not completely visible in the radiographs, generating the 3D spine model given only the encoded features for each vertebra separately could lead to inaccurate orientation.

Including the vertebral centroid coordinates in the global coordinate system provides the model with a holistic shape of the spine. For example, Figure 1 demonstrates lateral and anterior–posterior (AP) view radiographs of three different patients. The distance between the centroids determines the scale. If one fits a curve to all of the vertebral centroids, the orientation of each vertebra should be almost perpendicular to the curve at each vertebral centroid. Although defining the orientation of the vertebrae and the other constraints, such as inter-vertebral distance, needs accurate vertebral landmark detection on radiographs, we postulate a Multi Layer Perceptron (MLP) which estimates the 3D affine parameters, given only the vertebral centroids and the information extracted by the encoders.

Since the features calculated by the encoders for data samples in a batch are independent of each other, we need an intra-batch fusion mechanism to give the model a holistic view of the features from different spinal regions. We assume that the vertebrae in a batch are from the same spine and in order (we train the model in this way). For intra-batch fusion, we flatten the features from both encoders and also the down-scaled vertebral shape templates and concatenate them with the vertebral centroid coordinates, for all vertebrae. These inputs are fed into our affine subnetwork, which is an MLP.

For each vertebra, the affine subnetwork estimates four parameters: the scaling factor (*S*), and the rotation about each axis ($\theta_{x}$, $\theta_{y}$, $\theta_{z}$). After the transformation matrix is created, the corresponding affine vector field is calculated. We do not include translation, since the input image patches are extracted in such a way that the vertebral centroid is at a fixed location in the image patch.

(6) $$\theta_{x},\theta_{y},\theta_{z},S=AffineDecoder\left(x_{s},x_{c},y_{s},y_{c},{\vec{C}}^{\;v}\right),$$

(7) $$T_{affine}=R\left(\theta_{x}\right)\times R\left(\theta_{y}\right)\times R\left(\theta_{z}\right)\times T\left(S\right),$$

(8) $$R\left(\theta_{x}\right)=\left[\begin{array}{cccc} 1 & 0 & 0 & 0\\ 0 & \cos(\theta_{x}) & -\sin(\theta_{x}) & 0\\ 0 & \sin(\theta_{x}) & \cos(\theta_{x}) & 0\\ 0 & 0 & 0 & 1 \end{array}\right]$$

(9) $$R\left(\theta_{y}\right)=\left[\begin{array}{cccc} \cos(\theta_{y}) & 0 & \sin(\theta_{y}) & 0\\ 0 & 1 & 0 & 0\\ -\sin(\theta_{y}) & 0 & \cos(\theta_{y}) & 0\\ 0 & 0 & 0 & 1 \end{array}\right]$$

(10) $$R\left(\theta_{z}\right)=\left[\begin{array}{cccc} \cos(\theta_{z}) & -\sin(\theta_{z}) & 0 & 0\\ \sin(\theta_{z}) & \cos(\theta_{z}) & 0 & 0\\ 0 & 0 & 1 & 0\\ 0 & 0 & 0 & 1 \end{array}\right]$$

(11) $$T\left(S\right)=\left[\begin{array}{cccc} S & 0 & 0 & 0\\ 0 & S & 0 & 0\\ 0 & 0 & S & 0\\ 0 & 0 & 0 & 1 \end{array}\right]$$

(12) $${Affine}_{vf}=\tau \left(T_{affine}\right),$$

where the ${Affine}_{vf}$ is the affine vector field and τ() is the function to generate an affine vector field given the transformation matrix.

#### 2.3.3. Deformable Decoder

In parallel with affine parameter estimation for rough alignment between the template and target shapes, we synthesize the finer details. We design a subnetwork termed the “deformable decoder” for estimating local shape deformations. The deformable decoder is a fully convolutional network. The inputs to this network are the intermediate 3D latent representation calculated by the encoders with the down-scaled (the same size as 3D latent representation) shape template as an extra channel. Including the shape template as another input channel helps the model to infer the differences between the template and target shapes. The deformable decoder’s output is a 3D vector field with target volume resolution. Contrary to the affine decoder, which estimates the affine transformation parameters based on the entire batch, the deformable decoder is focused on single data samples in the batch, to consider the finer shape details for each sample,

(13) $${Deformable}_{vf}=DeformableDecoder\left(x_{s},x_{c},y_{s},y_{c}\right).$$

### 2.4. Learning

We train the model using the $\ell_{1}$ distance between the deformed templates and the target vertebral shapes in the voxel space. To regularize the deformations, we incorporate a smoothing term in the cost function. Solely using a regression loss leads to convergence to a local optimum in which a mean (or median) shape is predicted, especially in the highly varying regions of the vertebra such as the vertebral processes. We have,

(14) $$L_{total}=\alpha_{p}L_{\ell_{1}}+\alpha_{s}L_{smooth}$$

(15) $$L_{\ell_{1}}=||y-G\left(x_{s},x_{c},y_{s},y_{c},y_{t},{\vec{C}}^{\;v}\right)\circ y_{t}{\left|\right|}_{1}$$

(16) $$\begin{array}{c} L_{smooth}=\\ \frac{1}{X.Y.Z}\int_{0}^{X}\int_{0}^{Y}\int_{0}^{Z}||G\left(x_{s},x_{c},y_{s},y_{c},y_{t},{\vec{C}}^{\;v}\right){\left|\right|}_{1}dxdydz, \end{array}$$

where $\alpha_{p}$ and $\alpha_{s}$ are weights for the network prediction and smoothness terms respectively, and are fixed to $\alpha_{p}$ = 10 and $\alpha_{s}$ = 0.1. Note that y contains an integer value of {0,i}, where i∈{8,9,···,24} represents the vertebral index from T1 to L5. Constraining the network to predict the vertebral index implicitly requires it to learn relating the vertebral index to the shape as a prior.

In order to impose a constraint for locally smooth deformations and a minimum displacement solution for our registration problem, we add $L_{smooth}$ to penalize the $\ell_{1}-norm$ of the deformation field.

The network was implemented with the Pytorch framework on a Quadro P6000 GPU. It was trained until convergence using the Adam optimizer [32] with an initial learning rate of 0.0001.

### 2.5. Data

Recall that TransVert+ works with two data modalities: it is trained on DRRs extracted from CT images and is deployed on clinical radiographs.

#### 2.5.1. CT data

We employed two sets of data: First, a public dataset for lung nodule detection with 800 chest CT scans [33], and second, an in-house dataset with 154 spinal CT scans. Overall, we work with ∼12 K vertebrae split 5:1 forming the training and validation set and report 5-fold cross-validated results. Of note, ref. [33] is a lung-centred dataset, thus consisting of few lumbar vertebrae.

Usually in spine CT scans [34] some parts of the ribs and tissues in distance from the spine are excluded. Thus, these scans could not be used for generating DRRs that are similar in appearance to real radiographs. However, using lung CT scans result in better DRRs.

All of spinal CT scans were resampled to 1 mm resolution. The CT scans of our in-house dataset had ground truth segmentation and the CT scans in [33] were segmented using [35]. Next, an experienced neuro-radiologist approved the generated masks to consider only accurate ones for the study. Consequently, we excluded 50 cases from [33].

**Figure 6:**
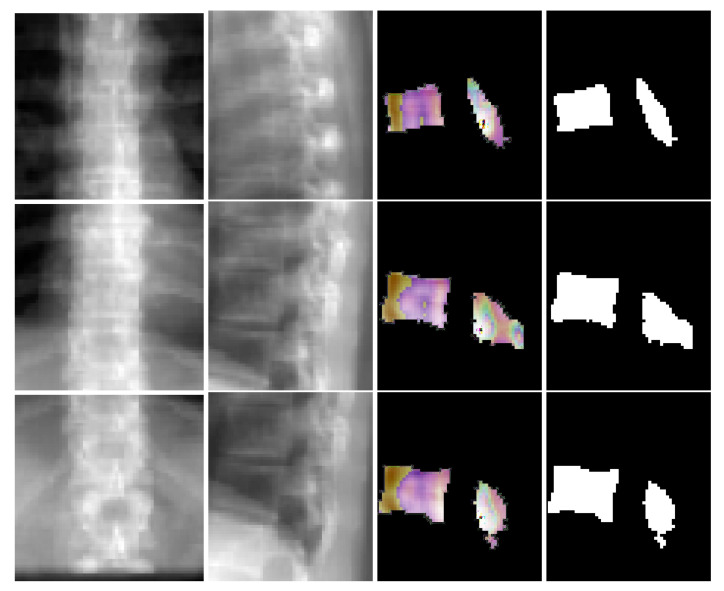
Visualization of coronal and sagittal image patches from three vertebrae. First and second columns are the coronal and sagittal image patches, third column shows one slice of the predicted deformation field and last column is the corresponding slice in the resulting shape.

**Figure 7:**
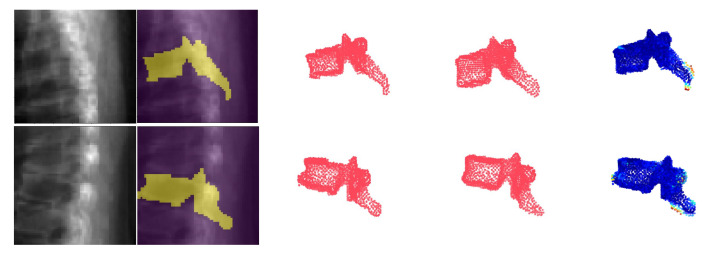
Shape modelling with TransVert+ on DRRs: The first column indicates the image input. The second and third columns visualize the ground truth (GT) vertebral mask, and the fourth visualizes the predicted 3D shape model. The last column shows an overlaid Chamfer distance map between point clouds of GT and prediction.

**Figure 8:**
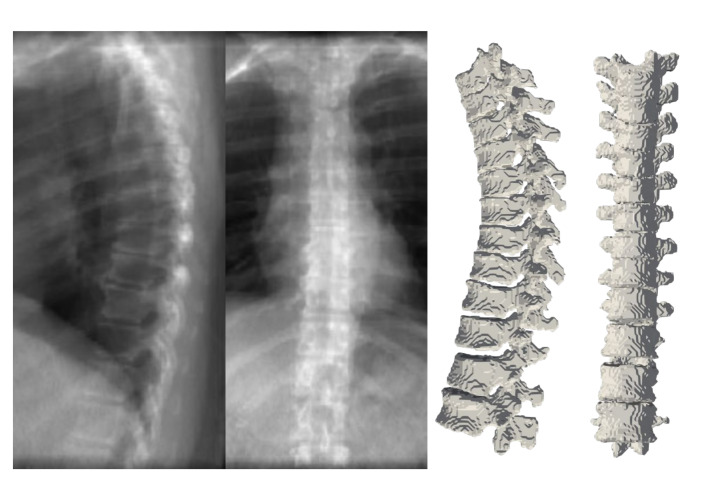
Orthogonal DRRs of a patient and reconstructed 3D spine model. DRRs are generated from CT scans and our model is trained to reconstruct the 3D spine shape from DRRs.

We employ a ray-casting approach [36] to construct DRRs from CT scan. In this method, we define lines from the radiation source (focal point) to every single pixel on the DRR image and calculate the integral of the CT intensities over these lines. In this simulation we assign (180cm) and (150cm) to the radiation source-to-detector distance and the source-to-object distance parameters respectively. Examples of patches extracted from DRRs are illustrated in Figure 6 and Figure 7. Also, a complete example of orthogonal DRRs is shown in Figure 8.

Once the sagittal and coronal DRRs are generated, the inputs for TransVert+ are constructed by extracting image patches of size 64 × 64 around each vertebral centroid. Similarly, the VOI-annotations were also extracted automatically from the projected segmentation mask.

#### 2.5.2. Clinical Radiographs

We validate TransVert+ on clinical, standing radiographs (pairs of lateral and anterior-posterior projections) acquired from 30 patients. Before deploying our TransVert+ model on the clinical radiographs, we resampled all radiographs to 1 mm resolution.

Image acquisition parameters such as the source-to-detector and source-to-object distances were similar to those used for DRR generation. We employed [37,38] to automatically generate the vertebral annotations on both views.

#### 2.5.3. Image Normalization

We trained TransVert+ on DRRs and tested it on real clinical radiographs. To enable a transfer of learning between these modalities, we need to normalize the intensities to a similar range. Therefore, we employ z-score normalization, i.e., $I=\left(I-\mu_{I}\right)/\sigma_{I}$, where $\mu_{I}$ and $\sigma_{I}$ are the mean and standard deviation of the image I, respectively.

### 2.6. Metrics

The Dice score is the most popular measurement for evaluating segmentation accuracy and measures the overlap between two binary images. However, the Dice score is a poor measure of segmentation accuracy when the shapes to be compared are not “blob-like”.

Another popular metric for segmentation evaluation is the Hausdorff distance. The Hausdorff distance between two segmentations represented as surface meshes is the maximum distance from vertices on the first mesh to the vertices on the second mesh.

The measurements, such as the Dice score, average many local errors measured at each voxel. Thus, a segmentation that is largely correct with a few major shape errors will have the same score as a segmentation that is only slightly wrong everywhere.

As mentioned before, we do not have the 3D ground truth for the clinical radiograph of a patient. We can acquire the 3D CT segmentation of the same patient. However, because of difference in spinal posture, the vertebral orientations are different and we cannot compare the 3D vertebrae reconstructed from clinical radiographs to the ones from CT using Dice score or Hausdorff distance. Thus, we desire a rotation-invariant metric to evaluate the model performance on clinical radiographs, for each individual vertebra.

The normalized weighted spectral distance (nWESD) [39,40] is a global shape measure based on heat trace analysis via the Laplace operator. The eigenvalues of the Laplacian of a shape are strongly connected to the shape’s geometric properties, such as its volume, surface area and mean curvature. The Laplace spectrum is invariant to isometric transformations (rigid transformations), and changes continuously as a shape’s boundary is transformed. Thus, because of rotational invariance, we can use this metric for comparing the 3D vertebral shapes reconstructed from the patient’s clinical radiographs to the vertebral shapes from their 3D CT segmentations.

The weighted spectral distance (WESD) between two binary segmentations $\Omega_{\lambda }$ and $\Omega_{\xi }$ is defined as:

(17) $$\rho \left(\Omega_{\lambda },\Omega_{\xi }\right)={\left[\sum_{n=1}^{\infty }{\left(\frac{|\lambda_{n}-\xi_{n}|}{\lambda_{n}\xi_{n}}\right)}^{p}\right]}^{1/p},$$

where $\lambda_{n}$ and $\xi_{n}$ denote eigenvalues of the segmentations $\Omega_{\lambda }$ and $\Omega_{\xi }$, and p∈R with p>d/2, *d* indicates the dimensionality of the binary segmentations.

The normalized WESD (nWESD) is derived using the fact that WESD converges as N→∞ (even though each eigenvalue spectrum is divergent) and is bounded above by $W(\Omega_{\lambda },\Omega_{\xi })$ (for details, see [39]). Therefore, the (finite) nWESD ${\bar{\rho }}^{N}\left(\Omega_{\lambda },\Omega_{\xi }\right)$ can be defined as:

(18) $${\bar{\rho }}^{N}\left(\Omega_{\lambda },\Omega_{\xi }\right)=\frac{\rho^{N}\left(\Omega_{\lambda },\Omega_{\xi }\right)}{W(\Omega_{\lambda },\Omega_{\xi })}\in \left[0,1\right)$$

(19) $$\rho^{N}\left(\Omega_{\lambda },\Omega_{\xi }\right)={\left[\sum_{n=1}^{N}{\left(\frac{|\lambda_{n}-\xi_{n}|}{\lambda_{n}\xi_{n}}\right)}^{p}\right]}^{1/p}.$$

### 2.7. Experiments

In order to analyze the contribution of various architectural components of the TransVert+ (fusion of sagittal and coronal views with shape prior, the affine decoder and a convolutional decoder to estimate the displacement field for each vertebrae) and to validate its performance on clinical radiographs, we propose three sets of experiments.

(1) We conducted an ablative study on the architectural choices including three steps: First, we evaluated the performance of the model working only with the affine decoder. Second, we trained the model solely with the deformable decoder to estimate the deformation fields. Third, we repeat the performance of our previous model TransVert, in which we did not incorporate the shape priors, but estimated the vertebral shapes directly in the voxel space without registration. Finally, we give results for TransVert+.
(2) We deployed the model on clinical radiographs to reconstruct 3D standing spine postures.
(3) We evaluated the model performance on clinical radiographs quantitatively. The performance evaluation in various settings was compared by computing the Dice coefficient, Hausdorff distance and normalized weighted spectral distance (nWESD) between the predicted 3D vertebral mask and the ground truth CT mask, where appropriate.

## 3. Results

The results of the ablation study are reported in Table 1. As we expected, the affine results are not better than TransVert, since the model roughly aligns the shape template to the target and the results lack shape details. Training the model with a deformable decoder performs better than the affine model. The deformable model achieves an average Dice score of 0.9405, close to that of the TransVert model (0.9426). In the Hausdorff distance metric, TransVert performs better but for the nWESD metric, the deformable model achieves a better score. Finally, our main TransVert+ model outperformed all of the previous models in all metrics.

Figure 6 depicts coronal and sagittal image patches from three vertebrae, mid-slice of the resulting estimated displacement field and vertebral shape. In the estimated displacement field the brighter colors indicate greater displacements. Figure 7 shows an example point cloud (with 2048 points) from the predicted and ground truth shapes, along with a point-wise Chamfer distance map. Observe that the vertebra’s posterior region (vertebral process) is hardly visible in the image inputs. In spite of this, TransVert+ was able to reconstruct the 3D shape of vertebral processes. To calculate the Chamfer distance map for each point in the reconstructed vertebra’s point cloud, the nearest point in the ground truth point cloud is found and the square of distance is depicted. Figure 8 illustrates a 3D spine reconstruction based on 2D DRRs.

**Figure 9:**
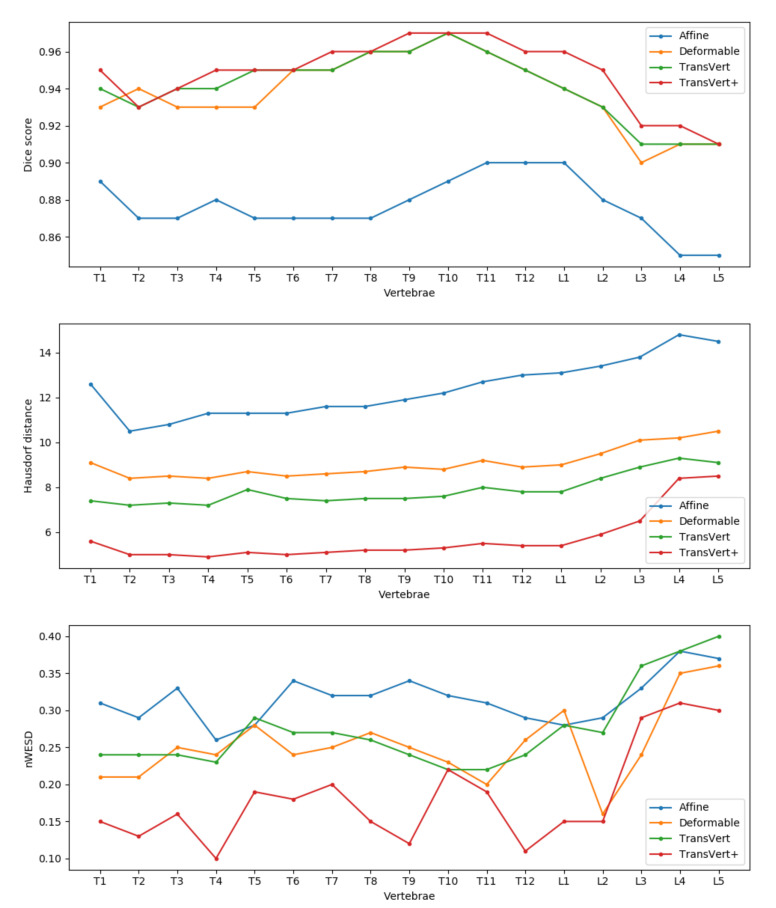
Vertebra-wise comparison of different architectures (Affine, Deformable, TransVert and TransVert+) using Dice scores, Hausdorff and nWESD distances. A higher Dice score indicates better performance. A lower Hausdorff and nWESD distance indicates better performance.

For a detailed comparison of the methods, in Figure 9 we report the mean Dice score, Hausdorff distance and nWESD distance for each vertebra. In general, TransVert+ outperforms almost all other models for each vertebra. According to this figure, the model performance drops in all metrics for lumbar vertebra.

### 3.1. 2D-to-3D Translation in Clinical Radiographs

**Figure 10:**
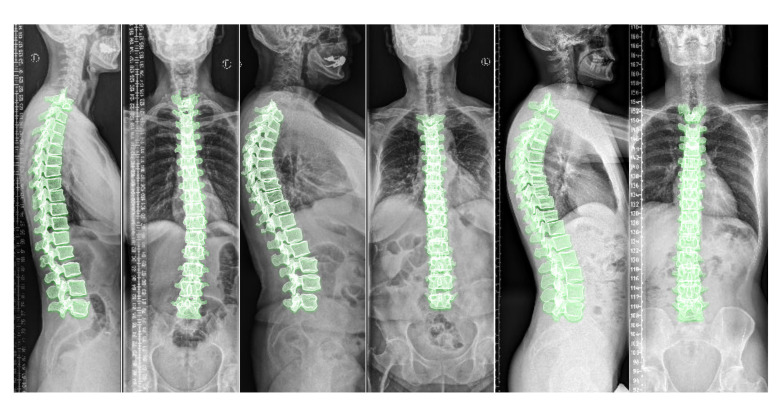
Full 3D spine models: 3D patient-specific spine models constructed from real clinical radiographs. Each sagittal and coronal view radiograph pair is from a different patient.

Figure 10 depicts the results of deploying TransVert+ for reconstructing the 3D, patient-specific posture of the upright standing spine. As stated, there is no 3D ground truth spinal model for the clinical radiographs. Observe the matched reconstruction of the 3D spine posture to the spinal posture in radiographs.

### 3.2. Quantitative Evaluation of Performance on Clinical Radiographs

**Figure 11:**
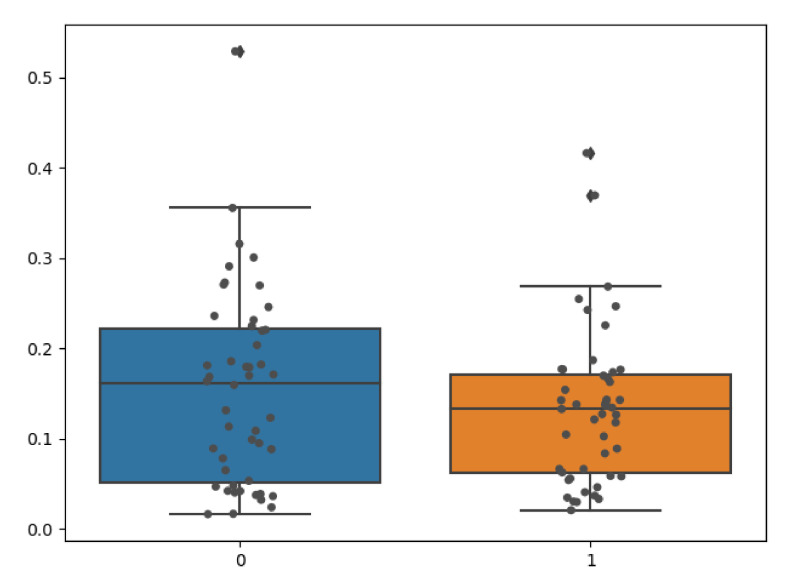
Comparing vertebral shapes to the ones predicted from radiographs using TransVert (Blue) and TransVert+ (Orange) using the nWESD metric. The mean nWESD metric for TransVert+ is lower than TransVert. For nWESD, lower is better.

We quantitatively evaluated the performance of our model on clinical radiographs in two patients for whom we have CT scans in addition to orthogonal standing radiographs. Using our TransVert model and the TransVert+ model proposed in this work, we generated the 3D shapes of the vertebrae given the 2D clinical radiographs. Then we compared each vertebra to the corresponding one from the CT scan segmentation masks, which refer to the same object but in a different orientation. Conventional metrics like the Dice score or Hausdorff are not applicable in this case, but we can use the nWESD metric as it is invariant to rigid transformations (including rotation). The resulting nWESD scores are demonstrated in Figure 11, where the mean of nWESD was 0.13 and 0.15 for TransVert+ and TransVert respectively. Similarly, the standard deviation of nWESD was 0.08 for TransVert+ and 0.11 for TransVert. These scores are calculated on a group of 32 vertebrae. Despite better values for TransVert+, the difference was not significant.

**Figure 12:**
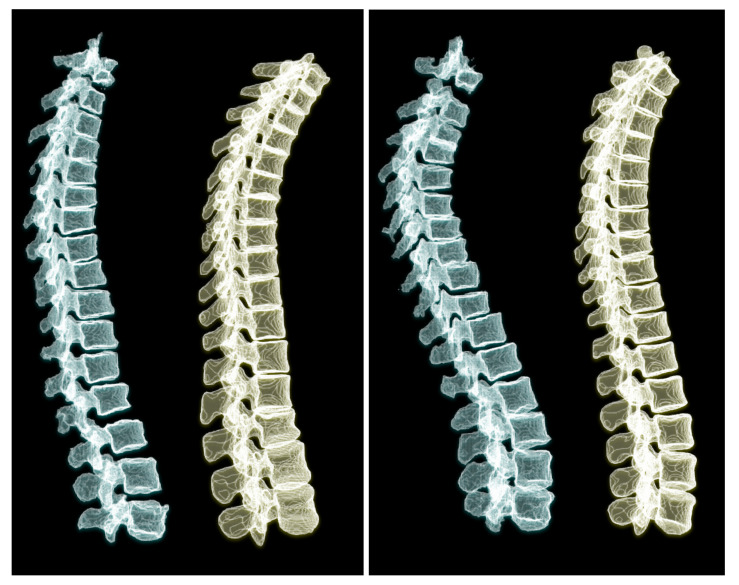
Comparing 3D reconstructions of the standing spinal posture from clinical radiographs (left, green) to the spinal posture of the same patient in lying-down position from CT imaging (right, yellow) in two different patients. In the upright-standing posture the spine is under natural weight bearing, which leads to a different spine curvature.

To appreciate the difference of spine posture in the standing and lying down positions, we illustrate the two cases we used in this experiment in Figure 12. The spine postures reconstructed from radiographs in upright standing position are depicted on the left and the ones from CT are depicted on the right side of the figure.

## 4. Discussion

The approach we proposed could be used to simulate the spinal posture in a standing position under weight bearing. In Table 1 we showed that our approach could improve overall model performance in terms of Dice score, Hausdorff distance and nWESD. However, more work is required to improve the results and overcome the limitations.

First, as explained in the results section (Figure 9) the model performance drops for lumbar vertebrae in all models because of few lumbar vertebrae in the lung-centered dataset we used. Second, the sagittal and coronal view radiographs might not be perfectly perpendicular in clinical settings; whereas, we generated DRRs with orthogonal projection angles.

Training the model with a dataset containing more scans with lumbar vertebrae, could improve the model performance in the lumbar region. As depicted in Figure 12, we compared the standing and lying down spine postures of two patients. Acquiring more annotated pairs of CT scans and radiographs of the same patient could enrich the results and provide more data for validation. We use a relatively large-scale dataset for training our model. Although the size and anatomical variations in patients helps the model learn the shapes and deformations, we believe that data augmentation by adding a small randomness to the projection angle in the simulated radiographs could teach the model this randomness in clinical radiographs.

## 5. Conclusions

We introduced TransVert+, a neural network architecture to reconstruct a full 3D spinal model from 2D orthogonal radiographs by deforming vertebral shapes. We proposed a supervised approach to train the model on synthetic data (DRRs) and transfer the trained model to radiographs. We demonstrated an improved performance of the model after incorporating shape priors into the model and separating the registration problem into affine and deformable registrations. We improved the state-of-the-art in three different metrics for this task. Finally, we successfully validated our model on real-world clinical radiographs and quantitatively compared the results to CT segmentations of the same patient for the first time.

## Figures and Tables

**Table 1:** Architectural ablation study: The performance progressively improves with the addition of each component. While TransVert outperforms the separate Affine and Deformable TransVert models in terms of Dice score and Hausdorff distance, including both of the Affine and the Deformable decoders in the TransVert+ model performs better than TransVert.

Setup	Dice	Hausdorff (mm)	nWESD
AffTransVert	0.8847	12.40	0.3181
DefTransVert	0.9405	9.08	0.2430
TransVert	0.9426	7.93	0.2779
TransVert+	0.9510	5.70	0.1797

## Data Availability

Not applicable.

## Abbreviations

The following abbreviations are used in this manuscript:

DRR	Digitally Reconstructed Radiographs
MRI	Magnetic Resonance Imaging
CT	Computed Tomography
nWESD	normalized Weighted Spectral Distance
VOI	Vertebra Of Interest
GT	Ground Truth

## References

[1] Dreischarf M., Shirazi-Adl A., Arjmand N., Rohlmann A., Schmidt H. (2016). Estimation of loads on human lumbar spine: A review of in vivo and computational model studies. J. Biomech..

[2] Akhavanfar M., Kazemi H., Eskandari A., Arjmand N. (2018). Obesity and spinal loads: A combined MR imaging and subject-specific modeling investigation. J. Biomech..

[3] El Ouaaid Z., Shirazi-Adl A., Plamondon A. (2014). Effect of changes in orientation and position of external loads on trunk muscle activity and kinematics in upright standing. J. Electromyogr. Kinesiol..

[4] Eskandari A., Arjmand N., Shirazi-Adl A., Farahmand F. (2017). Subject-specific 2D/3D image registration and kinematics-driven musculoskeletal model of the spine. J. Biomech..

[5] Löffler M.T., Jacob A., Scharr A., Sollmann N., Burian E., El Husseini M., Sekuboyina A., Tetteh G., Zimmer C., Gempt J. (2021). Automatic opportunistic osteoporosis screening in routine CT: Improved prediction of patients with prevalent vertebral fractures compared to DXA. Eur. Radiol..

[6] Bauer S., Hausen U., Gruber K. (2012). Effects of individual spine curvatures—A comparative study with the help of computer modelling. Biomed. Eng. Tech..

[7] Ketcha M., De Silva T., Uneri A., Jacobson M., Goerres J., Kleinszig G., Vogt S., Wolinsky J., Siewerdsen J. (2017). Multi-stage 3D–2D registration for correction of anatomical deformation in image-guided spine surgery. Phys. Med. Biol..

[8] De Silva T., Uneri A., Ketcha M., Reaungamornrat S., Kleinszig G., Vogt S., Aygun N., Lo S., Wolinsky J., Siewerdsen J. (2016). 3D–2D image registration for target localization in spine surgery: Investigation of similarity metrics providing robustness to content mismatch. Phys. Med. Biol..

[9] Humbert L., De Guise J.A., Aubert B., Godbout B., Skalli W. (2009). 3D reconstruction of the spine from biplanar X-rays using parametric models based on transversal and longitudinal inferences. Med. Eng. Phys..

[10] Aubert B., Vazquez C., Cresson T., Parent S., de Guise J.A. (2019). Toward automated 3D spine reconstruction from biplanar radiographs using CNN for statistical spine model fitting. IEEE Trans. Med. Imaging.

[11] Ying X., Guo H., Ma K., Wu J., Weng Z., Zheng Y. X2CT-GAN: Reconstructing CT from biplanar X-rays with generative adversarial networks. Proceedings of the IEEE Conference on Computer Vision and Pattern Recognition.

[12] Xie H., Yao H., Sun X., Zhou S., Zhang S. Pix2vox: Context-aware 3d reconstruction from single and multi-view images. Proceedings of the IEEE International Conference on Computer Vision.

[13] Xie H., Yao H., Zhang S., Zhou S., Sun W. (2020). Pix2Vox++: Multi-scale context-aware 3D object reconstruction from single and multiple images. Int. J. Comput. Vis..

[14] Bayat A., Sekuboyina A., Paetzold J.C., Payer C., Stern D., Urschler M., Kirschke J.S., Menze B.H. Inferring the 3D standing spine posture from 2D radiographs. Proceedings of the International Conference on Medical Image Computing and Computer-Assisted Intervention.

[15] Lee M.C.H., Petersen K., Pawlowski N., Glocker B., Schaap M. (2019). TETRIS: Template transformer networks for image segmentation with shape priors. IEEE Trans. Med. Imaging.

[16] Sokooti H., De Vos B., Berendsen F., Lelieveldt B.P., Išgum I., Staring M. Nonrigid image registration using multi-scale 3D convolutional neural networks. Proceedings of the International Conference on Medical Image Computing and Computer-Assisted Intervention.

[17] Milletari F., Rothberg A., Jia J., Sofka M. Integrating statistical prior knowledge into convolutional neural networks. Proceedings of the International Conference on Medical Image Computing and Computer-Assisted Intervention.

[18] Oktay O., Ferrante E., Kamnitsas K., Heinrich M., Bai W., Caballero J., Cook S.A., De Marvao A., Dawes T., O’Regan D.P. (2017). Anatomically constrained neural networks (ACNNs): Application to cardiac image enhancement and segmentation. IEEE Trans. Med. Imaging.

[19] Balakrishnan G., Zhao A., Sabuncu M.R., Guttag J., Dalca A.V. (2019). Voxelmorph: A learning framework for deformable medical image registration. IEEE Trans. Med. Imaging.

[20] Nosrati M.S., Hamarneh G. (2016). Incorporating prior knowledge in medical image segmentation: A survey. arXiv.

[21] Meng D., Keller M., Boyer E., Black M., Pujades S. Learning a statistical full spine model from partial observations. Proceedings of the International Workshop on Shape in Medical Imaging.

[22] Gajny L., Ebrahimi S., Vergari C., Angelini E., Skalli W. (2019). Quasi-automatic 3D reconstruction of the full spine from low-dose biplanar X-rays based on statistical inferences and image analysis. Eur. Spine J..

[23] Mirzaalian H., Wels M., Heimann T., Kelm B.M., Suehling M. Fast and robust 3D vertebra segmentation using statistical shape models. Proceedings of the 2013 35th Annual International Conference of the IEEE Engineering in Medicine and Biology Society (EMBC).

[24] Klinder T., Ostermann J., Ehm M., Franz A., Kneser R., Lorenz C. (2009). Automated model-based vertebra detection, identification, and segmentation in CT images. Med. Image Anal..

[25] Ruiz-España S., Domingo J., Díaz-Parra A., Dura E., D’Ocón-Alcañiz V., Arana E., Moratal D. Automatic segmentation of the spine by means of a probabilistic atlas with a special focus on ribs suppression. Preliminary results. Proceedings of the 2015 37th Annual International Conference of the IEEE Engineering in Medicine and Biology Society (EMBC).

[26] Schmidt S., Kappes J., Bergtholdt M., Pekar V., Dries S., Bystrov D., Schnörr C. Spine detection and labeling using a parts-based graphical model. Proceedings of the Biennial International Conference on Information Processing in Medical Imaging.

[27] Glocker B., Feulner J., Criminisi A., Haynor D.R., Konukoglu E. Automatic localization and identification of vertebrae in arbitrary field-of-view CT scans. Proceedings of the International Conference on Medical Image Computing and Computer-Assisted Intervention.

[28] Seifert S., Barbu A., Zhou S.K., Liu D., Feulner J., Huber M., Suehling M., Cavallaro A., Comaniciu D. (2009). Hierarchical parsing and semantic navigation of full body CT data. Proceedings of the Medical Imaging 2009: Image Processing.

[29] Zhan Y., Maneesh D., Harder M., Zhou X.S. Robust MR spine detection using hierarchical learning and local articulated model. Proceedings of the International Conference on Medical Image Computing and Computer-Assisted Intervention.

[30] Cai Y., Osman S., Sharma M., Landis M., Li S. (2015). Multi-modality vertebra recognition in arbitrary views using 3D deformable hierarchical model. IEEE Trans. Med. Imaging.

[31] Roy A.G., Navab N., Wachinger C. Concurrent spatial and channel ‘squeeze & excitation’in fully convolutional networks. Proceedings of the International Conference on Medical Image Computing and Computer-Assisted Intervention.

[32] Kingma D.P., Ba J. (2014). Adam: A method for stochastic optimization. arXiv.

[33] Armato S.G., McLennan G., Bidaut L., McNitt-Gray M.F., Meyer C.R., Reeves A.P., Zhao B., Aberle D.R., Henschke C.I., Hoffman E.A. (2011). The lung image database consortium (LIDC) and image database resource initiative (IDRI): A completed reference database of lung nodules on CT scans. Med. Phys..

[34] Liebl H., Schinz D., Sekuboyina A., Malagutti L., Löffler M.T., Bayat A., El Husseini M., Tetteh G., Grau K., Niederreiter E. (2021). A computed tomography vertebral segmentation dataset with anatomical variations and multi-vendor scanner data. Sci. Data.

[35] Sekuboyina A., Husseini M.E., Bayat A., Löffler M., Liebl H., Li H., Tetteh G., Kukačka J., Payer C., Štern D. (2021). VerSe: A vertebrae labelling and segmentation benchmark for multi-detector CT images. Med. Image Anal..

[36] Staub D., Murphy M.J. (2013). A digitally reconstructed radiograph algorithm calculated from first principles. Med. Phys..

[37] Bayat A., Sekuboyina A., Hofmann F., El Husseini M., Kirschke J.S., Menze B.H. Vertebral Labelling in Radiographs: Learning a Coordinate Corrector to Enforce Spinal Shape. Proceedings of the International Workshop and Challenge on Computational Methods and Clinical Applications for Spine Imaging.

[38] Sekuboyina A., Rempfler M., Valentinitsch A., Menze B.H., Kirschke J.S. (2020). Labeling Vertebrae with Two-dimensional Reformations of Multidetector CT Images: An Adversarial Approach for Incorporating Prior Knowledge of Spine Anatomy. Radiol. Artif. Intell..

[39] Konukoglu E., Glocker B., Criminisi A., Pohl K.M. (2013). WESD–Weighted Spectral Distance for Measuring Shape Dissimilarity. IEEE Trans. Pattern Anal. Mach. Intell..

[40] Konukoglu E., Glocker B., Ye D.H., Criminisi A., Pohl K.M. (2012). Discriminative segmentation-based evaluation through shape dissimilarity. IEEE Trans. Med. Imaging.

